# Modelling ischemia-reperfusion injury (IRI) *in vitro* using metabolically matured induced pluripotent stem cell-derived cardiomyocytes

**DOI:** 10.1063/1.5000746

**Published:** 2018-03-20

**Authors:** Alejandro Hidalgo, Nick Glass, Dmitry Ovchinnikov, Seung-Kwon Yang, Xinli Zhang, Stuart Mazzone, Chen Chen, Ernst Wolvetang, Justin Cooper-White

**Affiliations:** 1 Tissue Engineering and Microfluidics Group, Australian Institute for Bioengineering and Nanotechnology, The University of Queensland, Brisbane 4072, Australia; 2 Stem Cell Engineering Group, Australian Institute for Bioengineering and Nanotechnology, The University of Queensland, Brisbane 4072, Australia; 3 Laboratory for Respiratory Neuroscience and Mucosal Immunity, School of Biomedical Sciences, The University of Queensland, St. Lucia 4072, Australia; 4 Laboratory for Endocrinology and Metabolism, School of Biomedical Sciences, The University of Queensland, St. Lucia 4072, Australia; 5 School of Chemical Engineering, The University of Queensland, Brisbane 4072, Australia; 6 CSIRO, Manufacturing Flagship, Biomedical Manufacturing Program, Clayton, Victoria 3810, Australia

## Abstract

Coronary intervention following ST-segment elevation myocardial infarction (STEMI) is the treatment of choice for reducing cardiomyocyte death but paradoxically leads to reperfusion injury. Pharmacological post-conditioning is an attractive approach to minimize Ischemia-Reperfusion Injury (IRI), but candidate drugs identified in IRI animal models have performed poorly in human clinical trials, highlighting the need for a *human* cell-based model of IRI. In this work, we show that when we imposed sequential hypoxia and reoxygenation episodes [mimicking the ischemia (I) and reperfusion (R) events] to immature human pluripotent stem cell-derived cardiomyocytes (hPSC-CMs), they display significant hypoxia resistance and minimal cell death (∼5%). Metabolic maturation of hPSC-CMs for 8 days substantially increased their sensitivity to changes in oxygen concentration and led to up to ∼30% cell death post-hypoxia and reoxygenation. To mimic the known transient changes in the interstitial tissue microenvironment during an IRI event *in vivo*, we tested a new *in vitro* IRI model protocol that required glucose availability and lowering of media pH during the ischemic episode, resulting in a significant increase in cell death *in vitro* (∼60%). Finally, we confirm that in this new physiologically matched IRI *in vitro* model, pharmacological post-conditioning reduces reperfusion-induced hPSC-CM cell death by 50%. Our results indicate that in recapitulating key aspects of an *in vivo* IRI event, our *in vitro* model can serve as a useful method for the study of IRI and the validation and screening of human specific pharmacological post-conditioning drug candidates.

## INTRODUCTION

Current clinical practice aimed at limiting injury following STEMI involves prompt management with thrombolytic therapy or primary percutaneous coronary intervention (PPCI).^1,2^ These therapeutic interventions however cause ischemia reperfusion injury (IRI) that exacerbates myocardial cell death, which is one of the main contributors to coronary heart disease (CHD).^1,3^ IRI has been widely studied in *in vivo* and *in vitro* animal models,^4,5^ revealing important roles for local acidification, autophagy, reactive oxygen species (ROS)-production, mitochondrial-induced cell death, and associated adenosine triphosphate (ATP)-decline.^4–6^ Pharmacological post-conditioning (PPC) in previous studies has identified reoxygenation protocols and compounds that, when administered after the ischemic event, minimise IRI-induced myocardial injury.^7–9^ However, a large number of potential PPC-compounds identified using these models have largely failed to translate into successful human clinical trials.^7^ Intrinsic differences between human and animal heart physiology and/or differences in experimental design are the major source of error. To investigate the molecular mechanisms underlying IRI and discover better PPC drugs, a human cell-based model that recapitulates the transient microenvironmental changes experienced by cardiomyocytes during an IRI is highly desirable.^10^

Human pluripotent stem cell-derived cardiomyocytes (hPSC-CMs) have enabled human disease modelling and screening of pharmacologically relevant drugs *in vitro.*^11–14^ However, hPSC-derived cardiomyocytes resemble a more fetal-like state, which relies on glycolysis and therefore allows better survival in low oxygen environments. Due to this limitation, hPSC-derived cardiomyocytes have thus not been used to study IRI to date.^11,15–19^ Immediately after birth, cardiomyocytes display a rapid increase in mitochondrial mass and an accompanying metabolic shift from anaerobic glycolysis to mitochondrial β-oxidation of fatty acids,^20–22^ and this increased reliance on mitochondrial respiration is thought to underlie the susceptibility of adult cardiomyocytes to a hypoxic insult and reperfusion.^20,23–25^

Herein, we detail a novel *in vitro* human cell-based IRI model that mimics the known physiological changes [in terms of temporal transients in oxygen concentration, local pH, and glucose (glycogen) availability] experienced by cardiomyocytes during an IRI event. We first show that, post-induction of hPSCs to a cardiomyocyte fate, a short period of metabolic shift from glycolysis to oxidative phosphorylation of fatty acids is critical in promoting cardiomyocyte maturation and rendering the hPSC-CMs sensitive to sequential exposure to hypoxia and reoxygenation—our first pass at mimicking an IRI insult. We next show that matured hPSC-CM sensitivity to such an insult is further enhanced by recapitulating other microenvironmental pathological conditions present during the ischemic episode in the myocardium, resulting in the creation of a new *in vitro* IRI model. We finally demonstrate that this new *in vitro* IRI model can be used as a simple and scalable *in vitro* screening platform for the validation of known and discovery of novel PPC drugs.

## RESULTS

### Immature human pluripotent stem cell-derived cardiomyocytes are resistant to a simplified model of an IRI event

To model an ischemic-reperfusion insult *in vitro*, H9-NCX1^+^ cardiomyocytes (hereafter NCX1^+^-CMs) were exposed to sequential periods of hypoxia and reoxygenation. First, maintenance media were exchanged with oxygen depleted, glucose-free medium, and the cells were incubated in a 0% oxygen incubator for 2 h—this constitutes the ischemic event.^28^ To model the reperfusion event, the NCX1^+^-CMs were next exposed to oxygenated glucose-rich medium and transferred to a normoxic incubator for 1 or 4 h. This temporal sequence of events mimics a best-case intervention scenario for acute myocardial infarction (AMI) patients. Under this imposed simplified mimic of an IRI event, quantification of cell death by cleaved-Caspase 3 immunostaining revealed the absence of cell death after hypoxia-reoxygenation and persistence of beating activity regardless of the timeframe of hypoxia (1–4 h) (supplementary material, Fig. 1). These findings were expected to be the result of newly derived hPSC-CMs resembling fetal cardiomyocytes, a cell type able to cope much better with low oxygen environments than CMs of the adult myocardium.^29^

**FIG. 1.**
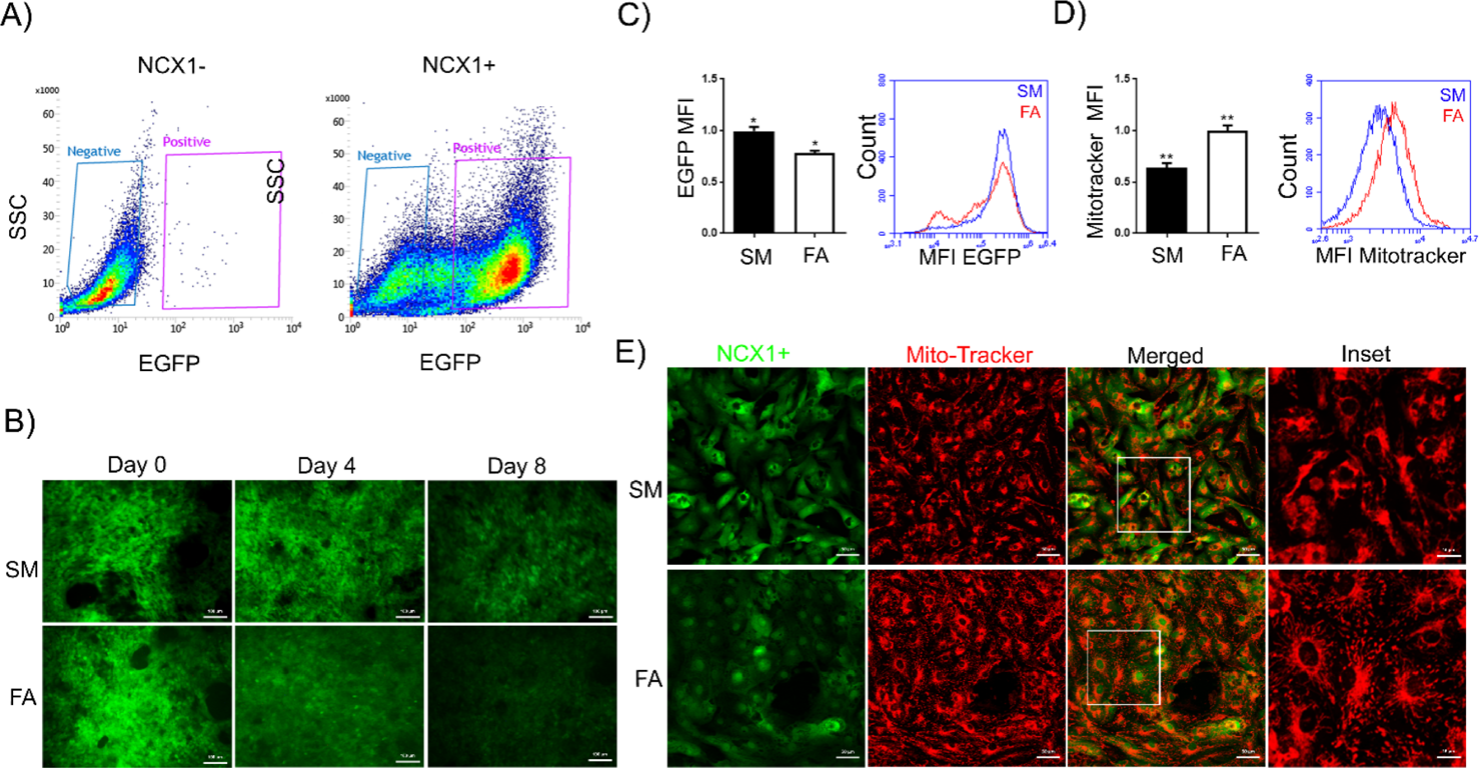
Immature NCX1^+^-CMs are unaffected by hypoxia-reoxygenation requiring maturation by modulation of substrate availability. (a) FACS dot plots for NCX1^+^-CM sorting based on GFP expression. (b) and (c) Decreased GFP expression in NCX1^+^-CMs during 8 days in Standard media (SM) and Fatty Acid media (FA) treatment (quantified by FACS) supports cardiomyocyte maturation with FA treatment. The Blue Mean Fluorescence Intensity (MFI) peak represents the SM cardiomyocytes, and the Red MFI peak represents the FA treated cells. (d) and (e) Mitotracker labelling of NCX1^+^-CM cultures following 8 days of culture in SM and FA media (quantified by FACS) revealed increased cytoplasmic mitotracker staining and a 40% increase in mitochondrial mass in FA treated cultures (n = 3).

### Metabolic maturation of human pluripotent stem cell derived cardiomyocytes

We hypothesized that hPSC-CMs with metabolic phenotypes comparable to postnatal cardiomyocytes would be more susceptible to IRI-induced cell death. To this end, we subjected immature hPSC-CMs to a metabolic maturation protocol previously reported to increase CM respiration rates.^30^ Depriving hPSC-CMs of glucose while providing galactose promotes mitochondrial beta-oxidation of long-chain fatty acids (palmitate and oleic acid), which mimics the metabolic switch from glycolysis to oxidative phosphorylation observed after birth,^31,32^ and fosters cardiomyocyte metabolic maturation.^30^ Immature green fluorescent protein (GFP)-sorted NCX1^+^-CM cells were cultured in either standard glucose-containing maintenance media [hereafter Standard Media (SM)] or glucose free, galactose supplemented fatty acid (palmitate and oleic)-rich media [hereafter fatty acid (FA)] for 8 days. Because expression of Sodium-Calcium exchanger 1 (NCX1) decreases during CM maturation,^33^ we used GFP expression driven by a cardiomyocyte-specific NCX1 promoter as an initial readout of maturation. Immunofluorescence [Fig. 1(b)] and flow cytometry [Fig. 1(c)] analyses revealed a significant reduction in mean fluorescence intensity (MFI) of GFP expression in CMs exposed to the FA culture condition as compared to the standard medium. Whilst the immunofluorescence images confirm this clear reduction in GFP expression as a result of exposure to the FA media, from our flow cytometry data, it appears that the reduction in overall MFI is supported by the appearance of subpopulations of cells that have varying levels of strongly decreased fluorescence, whereas some cells remain similar to those cells in SM, suggesting that across the population of cells, not all cells are transitioning at the same time, likely due to differences in the initial cell state. Shortly after birth, postnatal cardiomyocytes undergo an increase in mitochondrial mass and intracellular localization of mitochondria also shifts from the peri-nuclear space to a one that spreads across the cytoplasm. *In vivo* CM maturation and PGC1ɑ-enforced maturation^21^ of hPSC-derived CMs *in vitro* are similarly accompanied by an increase in the mitochondrial content and cytoplasmic relocalization of mitochondria. To evaluate if our maturation protocol is capable of inducing an increase in mitochondrial mass and the re-localization phenotype in hPSC-CMs, we next labelled SM- and FA-treated cultures with mitotracker-FM. FA-treated cardiomyocytes were replated onto aligned microgrooved substrates to promote cell elongation for visualisation of mitochondrial intracellular localization. Mitochondria occupied a large volume of the intracellular space (supplementary material, Fig. 2 and Video 2). Flow cytometry analysis showed that there was a 40% increase in mitochondrial mass in FA-treated CMs [Fig. 1(d)]. In addition, immunofluorescence staining showed that mitochondria are localized to the perinuclear space in SM-treated CMs, while in FA-treated CMs, larger mitochondria are arranged throughout the cytoplasm [Fig. 1(e)].

**FIG. 2.**
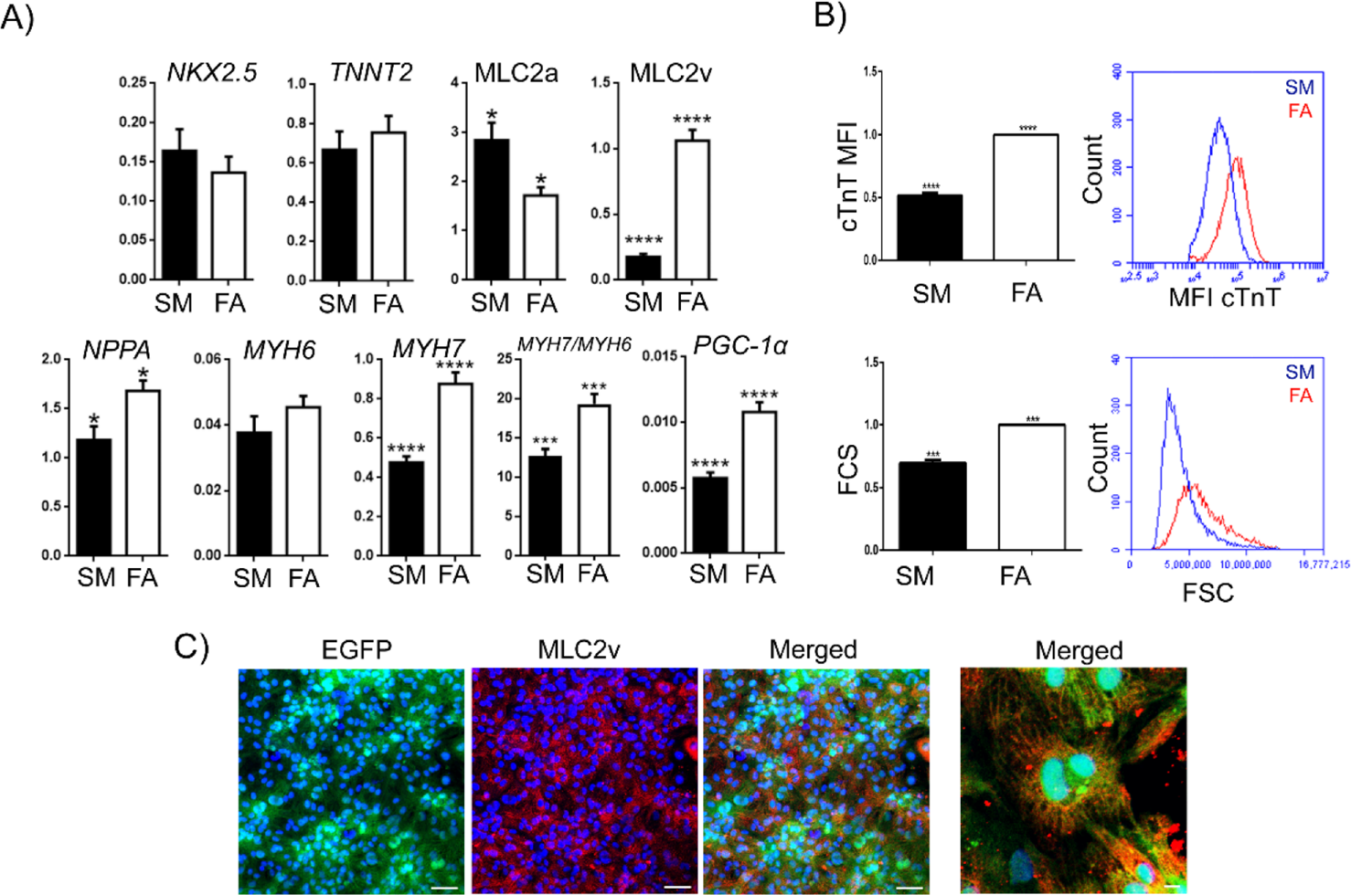
Characterisation of changes in gene and protein expression of NCX1^+^-CM phenotypic maturation. (a) Gene expression analysis of SM vs FA-treated NCX1^+^-CMs by qPCR (n = 3) after 8 days of metabolic maturation, Gene expression relative to *GAPDH* (ΔCt). (b) FACS-based quantification of cTnT expression and size of SM versus FA-treated NCX1^+^-CMs (n = 3) after 8 days in culture. (c) Immunofluorescence detection of MLC2v (red) expression in FA-treated NCX1^+^-CMs (far RHS—enlargement of the merged image).

We next examined mRNA expression of a set of sarcomeric cardiac proteins in SM and FA-treated NCX1^+^-CMs by quantitative polymerase chain reaction (qPCR). In the FA-treated cultures, we observed significantly increased expression of *MHY7* and *MLY2* (MLC2v) and downregulation of *MLY7* (MLC2a) but no significant differences *in NKX-2.5, MYH6*, *or TNNT2* (cTnT) expression between SM and FA-treated CMs [Fig. 2(a)]. The elevated MLC2v expression^34^ and increased ratio between β-MHC (*MYH7*) and α-MHC (*MYH6*)^35^ are consistent with a maturing CM phenotype.^17^ FA-treated cells also exhibited robust upregulation of *PGC-1a* mRNA [Fig. 2(a)], a gene directly involved in mitochondrial biogenesis and maturation of hPSC-CMs.^21^ The cardiac chamber specification marker natriuretic peptide type A (NPPA) is also upregulated in FA-treated CMs compared to SM-treated cells. Flow cytometric analysis revealed an increase in the cardiomyocyte size (Forward Scatter) in FA-treated cultures [Fig. 2(b)], as expected for physiological hypertrophy in maturing cardiomyocytes, and elevated sarcomeric protein expression. We further found increased expression of MLC2v protein in FA-treated cultures that persisted up to 24 days as indicated by immunofluorescence staining [Fig. 2(c)].

**FIG. 3.**
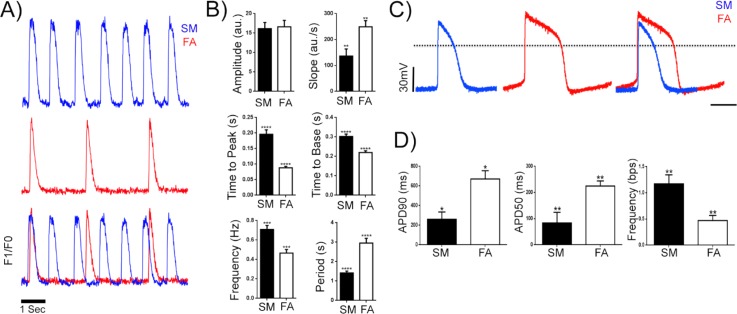
Characterisation of functional maturation of FA-treated hiPSC-derived cardiomyocytes. (a) Representative calcium transient in SM (blue) vs FA (red) treated cardiomyocytes measured by confocal line scanning (100 Hz) of hiPSC GCaMPG6f cell lines. (b) Quantification of calcium transient parameters in SM and FA-treated cardiomyocytes (n = 20). (c) Representative ventricular-like action potential of whole cell patch clamp recordings of SM vs FA treated CMs after 8 days of *in vitro* maturation. SM is represented as the blue trace, and FA is represented as the red trace. The dotted line represents the 0 mV baseline. (d) Quantification of electrophysiological parameters of cardiomyocytes with ventricular-like action potential from each of the SM and FA treated populations (n = 3 for each) from patch clamp recordings.

Since adult cardiomyocytes have a slower beating frequency than fetal cardiomyocytes, we quantified the beating frequency of SM and FA-treated cardiomyocytes by intracellular calcium transients. To this end, we employed a footprint-free human induced pluripotent stem cell (iPSC) line C32 that was transduced with a lentivirus carrying the GCaMP6f calcium biosensor^36^ (see Methods section and real-time output in supplementary material, Video 3). Calcium flux analysis revealed that FA-treated CM cultures exhibited clear differences in intracellular calcium handling [example traces shown in Fig. 3(a), line scan images of GCaMP6f mature single cardiomyocytes in supplementary material, Fig. 3), with an increase in the rising slope, a shorter time-to-peak, a shorter time-to-base, and a decrease in beating frequency from 0.9 beats/s vs 0.45 beats/s, but no change in the amplitude [Fig. 3(b)]. Collectively, the features observed in the calcium transients in FA-treated CMs are consistent with a more mature CM phenotype than those observed in SM-treated CMs.

To confirm these data, we collected electrophysiological recordings from SM and FA-treated NCX1^+^-CM cardiomyocytes. Figure 3(c) shows traces of patch-clamp recordings comparing SM and FA-treated cardiomyocytes with ventricular electrophysiological characteristics. FA-treated cardiomyocytes exhibit higher action potential depolarization (APD50 and APD90) and lower frequency compared to the SM-treated cardiomyocytes [Fig. 3(d)].

We conclude that, compared to SM-treated, FA-treated CMs exhibit mitochondrial, cell size, protein expression, mRNA expression, calcium handling, and membrane action potential characteristics consistent with a mature cardiomyocyte phenotype.

### Mimicking physiological changes in pH and glucose availability during IRI render matured hPSC-derived cardiomyocytes susceptible to ischemia-reperfusion *in vitro*

We next wished to assess whether the degree of maturation observed with FA-treated CMs was now sufficient to render the cells sensitive to our previously applied *in vitro* mimic of an IRI insult. However, in contemplating our previous IRI experiments, we also considered that there are other important changes in the tissue microenvironment during an *in vivo* IRI. Following an ischemic insult, there is a reversal from oxidative phosphorylation to anaerobic glycolysis, leading to a decrease in intracellular pH.^28^ To counteract this change, the transmembrane Na^+^/H^+^ exchanger extrudes excess H^+^ into the extracellular space, in exchange for Na^+^, lowering the local pH within interstitial tissue. The Na^+^/Ca^2+^ exchanger, forced to operate in the reverse mode to balance Na^+^, is now pressed to maintain intracellular Ca^2+^ levels, which leads to a rapid accumulation of intracellular Ca^2+^ that subsequently contributes to activation of proteases^37^ and increased apoptosis.^38^ We hypothesized that within standard culture ware environs, the cell surface area to media volume ratio of static culture plates would buffer the expected change in the extracellular concentration of (extruded) H^+^ following any mimicked IRI event *in vitro*, making it difficult to mimic known interstitial tissue microenvironmental changes.

**FIG. 4.**
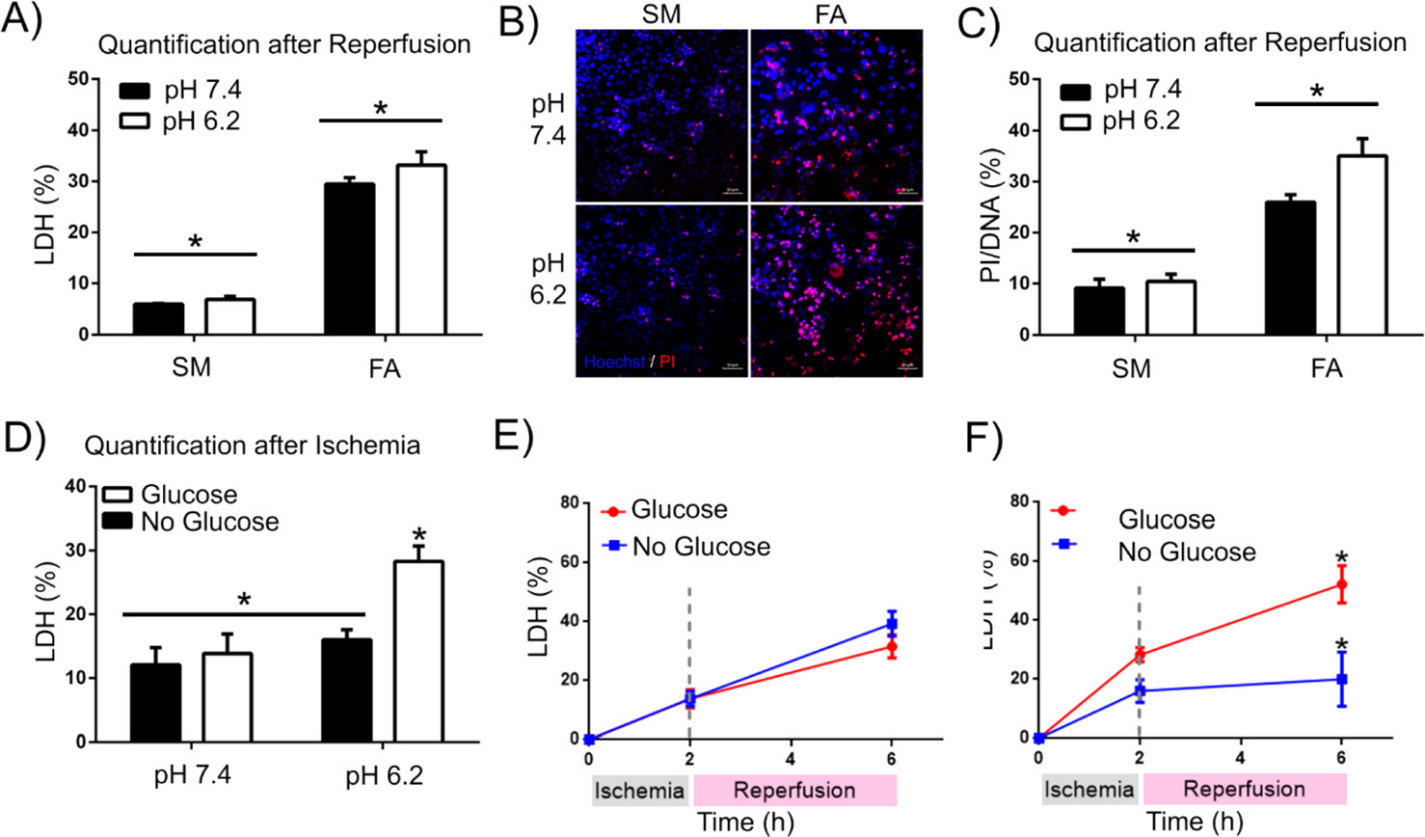
Effect of pH and glucose levels during IRI on cell death of matured cardiomyocytes. (a) Cardiomyocyte death [% Lactate dehydrogenase (LDH) release] after an ischemia-reperfusion episode (2 h of Ischemia + 4 h of Reperfusion) of SM and FA treated cells at pH 7.4 and 6.2. (b) Fluorescence images and (c) quantification thereof of the amount of cardiomyocyte death (by PI/Hoechst staining) after hypoxia-reoxygenation (2 h of Ischemia + 4 h of Reperfusion) of SM and FA-treated cells at pH 7.4 and 6.2. (d) LDH release quantification on matured cardiomyocytes at pH 7.4 and 6.2 in the presence of 5.5 mM glucose or the absence of glucose after an ischemic episode (2 h only). (e) and (f) Total cell death quantification with LDH release of matured cardiomyocytes after ischemia-reperfusion (2 + 4 h) in the presence of 5.5 mM glucose or the absence of glucose during the ischemic event, at pH 7.4 (e) and pH 6.2 (f) (n = 3).

In order to recapitulate robust cardiomyocyte cell death post an IRI event, we thus investigated the impact of a low media pH (of 6.2 instead of 7.4) during the IRI event. When we compared cardiomyocyte cell death, based on lactate dehydrogenase (LDH) release [Fig. 4(a)] and propidium iodide (PI) staining [Figs. 4(b) and 4(c)], in SM and FA-treated CMs subjected to an IRI episode at pH 7.4 or 6.2, we observed that while FA-treated CMs exhibited increased cell death, as compared to standard cultured cardiomyocytes at pH 7.4 (7% LDH and 10% PI for standard versus 29% LDH and 25% PI for matured cardiomyocytes), this was more pronounced at pH 6.2 for FA-treated/matured cardiomyocytes (30% LDH and 35% PI).

It was thus clear that FA-treated CMs were more susceptible to IRI-induced cell death; however, based on the known *in vivo* microenvironment surrounding CMs during an ischemic event, we next questioned the potential impact of the glucose-free medium during the hypoxia period in ischemia models^28^ on the observed outcomes. We hypothesized that the absence of glucose in the media during ischemia might induce activation of autophagy,^39^ preclude lactate-induced intracellular acidification, and/or lead to a drop in ATP (required for apoptotic cell death), ultimately resulting in a reduction in hypoxia-reoxygenation induced cell death of cardiomyocytes.

To test this hypothesis, we imposed hypoxia on FA-treated CMs for 2 h at pH 6.2 or 7.4 in the absence or the presence of 5.5 mM glucose and quantified cell death by LDH release immediately thereafter (no reoxygenation step). We found that at pH 6.2, the addition of glucose increased cell death 1.7-fold from 15% to 28%, but that at pH 7.4, the presence of glucose did not significantly impact hypoxia-induced cardiomyocyte cell death [Fig. 4(d)]. When we next subjected FA-treated cardiomyocytes to 2 h of hypoxia in the absence or the presence of glucose at pH 6.2 followed by 4 h of reoxygenation at pH 7.4 (to mimic reperfusion conditions), we observed that at physiological extracellular pH (7.4) levels, significantly reduced cell death and no differences were observed [Fig. 4(e)]. Applying the same hypoxic treatment, 50% of cardiomyocytes exposed to glucose-containing medium during hypoxia died, whereas those incubated in glucose-free medium during hypoxia exhibited only 25% cell death [Fig. 4(f)]. Further analysis showed an increased LC3B-II protein expression after only 2 h of glucose depletion, indicative of activation of autophagy (supplementary material, Fig. 4).

Collectively, these data demonstrate that metabolic maturation of cardiomyocytes is essential for modelling ischemia-reperfusion injury with hPSC-derived cardiomyocytes. Furthermore, they show that during the ischemic event, both physiological amounts of glucose and acidosis are required to mimic the levels of IRI induced cardiomyocyte cell death *in vivo*.

### Modelling pharmacological post-conditioning with matured hPSC-derived CMs and a new approach to mimicking IRI *in vitro*

**FIG. 5.**
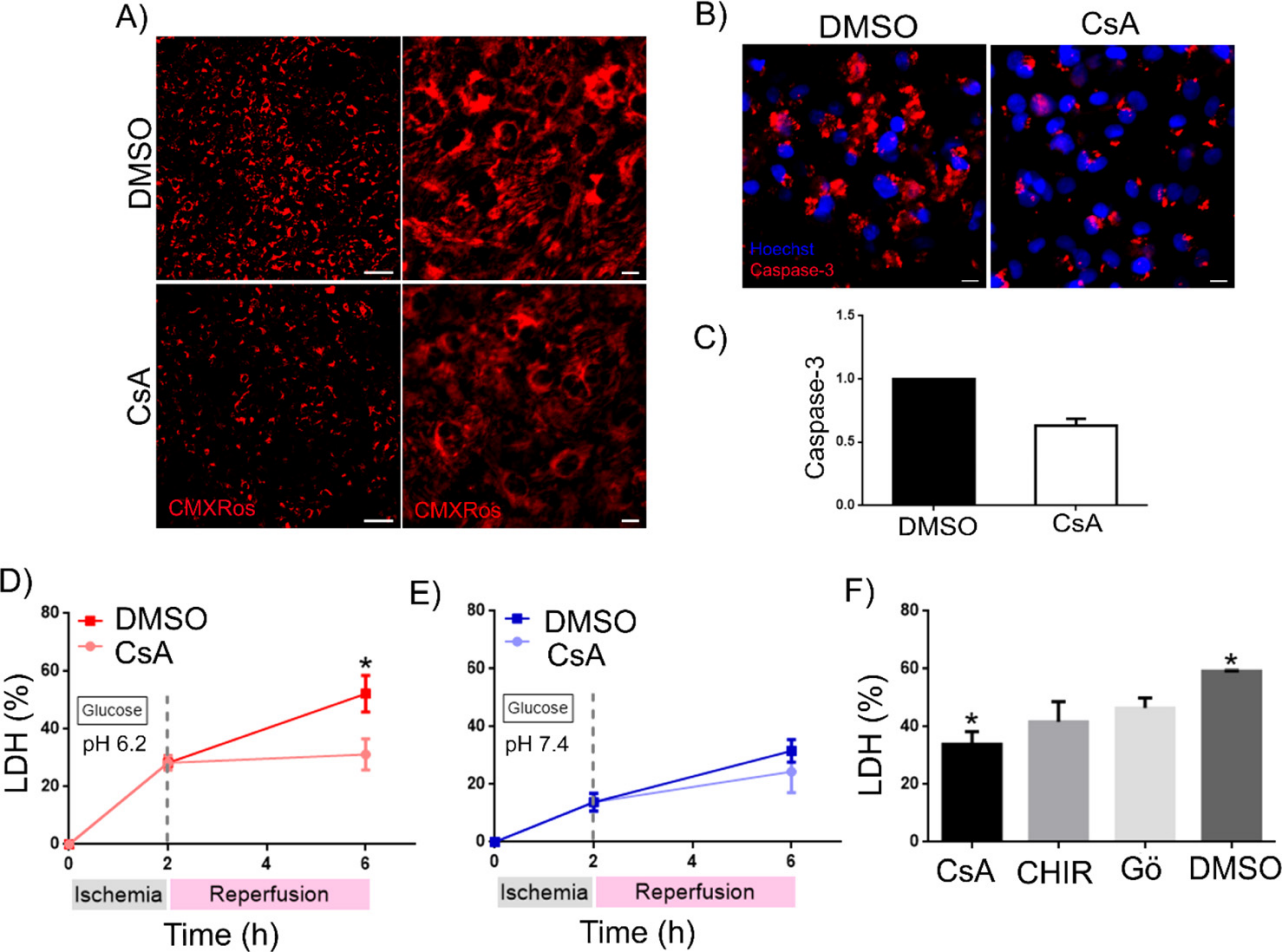
Pharmacological post-conditioning with mTPT upstream modulators in our IRI model confirms reduced cardiomyocyte death. (a) MitoTracker CMXRos staining of FA-treated cardiomyocytes subjected to IRI (2 h of ischemia followed by 4 h of reperfusion) with PPC or without (i.e., vehicle only) the addition of 1 *μ*M CsA during the re-oxygenation period. (b) and (c) Caspase-3 staining and quantification of Caspase-3 labelling of FA treated cardiomyocytes subjected to the IRI with the PPC model (n = 3), with results normalised to DMSO. (d) and (e) LDH quantification of FA-treated cardiomyocytes after IRI (6 h = 2 h of ischemia with glucose and 4 h of reperfusion) at pH 6.2 (d) or pH 7.4 (e) during ischemia and thereafter treated with vehicle (DMSO) or Cyclosporine A (1 *μ*M) during 4 h of reperfusion (n = 3). (f) Effect of PPC with CHIR99021 and Gö6976 [compared to vehicle (DMSO) or Cyclosporine A (1 *μ*M)] on LDH levels of FA-treated cardiomyocytes post-IRI (6 h = 2 h of ischemia at pH 6.2 and with glucose and 4 h of reperfusion).

PPC of the heart can be as effective as ischemic mechanical post-conditioning to reduce the infarct size after an ischemia-reperfusion episode *in vivo* and CM death *in vitro.*^2,40,41^ For instance, Cyclosporin A (CsA) has been successfully used for PPC and effectively reduces cell death and final infarct area.^9^ To validate our hPSC-CM IRI model as a useful platform for discovering and testing novel PPC drugs, we tested whether the reported beneficial effects of reduced mitochondrial permeability transition pore (mPTP) opening by CsA can be recapitulated *in vitro*. To test this, we applied 2 h of hypoxia at physiological glucose concentration at pH 6.2 and 7.4 or in the absence of glucose at pH 6.2 and pH 7.4 and thereafter included CsA during 4 h of reoxygenation at high glucose and pH of 7.4 or 6.2. Cardiomyocytes exposed to IRI in the presence of low glucose at pH 6.2 and treated with CsA during the reoxygenation period displayed lower levels of ROS production [as measured with MitoTracker CMXRos [Fig. 5(a)]. CsA treated CMs also displayed significantly lower expression of cleaved-caspase-3 [Figs. 5(b) and 5(c)], compared to cells treated with vehicles, and this treatment resulted in no further cell death {LDH release [Fig. 5(d)]} beyond that imparted by the hypoxic insult. CsA treatment during reperfusion effectively reduced cell death by 41%, as compared to vehicle treated cardiomyocytes at the same conditions [Fig. 5(d)]. Interestingly, when the hypoxic and reoxygenation events were performed at physiological pH of 7.4, PPC with CsA had no significant reduction effect on cell death [Fig. 5(e)], emphasizing the critical role of acidification and possible direct effects on mitochondrial membrane integrity. The mitochondrial ATP-sensitive K^+^ channel (mK_ATP_ channel) and GSK3β are key mediators for mPTP opening, acting upstream of CsA.^42^ To further test our model, we show that treatment with small molecules Gö 6976 [inhibitor of protein kinase C (PKC) (which acts on mK_ATP_)] and CHIR99021 (a GSK3β inhibitor) also showed an apparent reduction in LDH release compared to vehicles [Fig. 5(f)]. These results validate that our *in vitro* model recapitulates *in vivo* cellular events during IRI and could be a valuable tool to assess efficacy of small molecules in PPC.

## DISCUSSION

In this study, we have established a new human cell-based *in vitro* model of IRI using hPSC-derived cardiomyocytes for the study of pharmacological post-conditioning. We identified that maturation of cardiomyocytes, lower pH, and availability of glucose during ischemia are indispensable for the establishment of a representative *in vitro* model for ischemia-reperfusion studies.

In agreement with others,^37,43^ we found that switching hPSC-derived cardiomyocytes from glucose-based medium to a galactose/fatty acid-based medium for 8 days is sufficient to foster maturation of these hPSC-derived cardiomyocytes. Maturation was evidenced by (i) decreased expression of NCX1 channels, (ii) increased cell size, (iii) increased mitochondrial mass, (iv) relocalisation of mitochondria, (v) increased expression of the mitochondrial biogenesis marker *PGC1-α*, (vi) acquisition of functional characteristics of more mature cardiomyocytes, and (vii) mRNA and protein expression changes.

Whilst FA-treated cardiomyocytes displayed decreased viability after our initial model of an IRI episode, as compared to SM-treated cardiomyocytes, this was enhanced by our modified IRI model where we expose the cells to low pH and glucose during the hypoxia period.

Myocardial ischemia triggers a rapid decline in ATP and increases adenosine monophosphate (AMP)/ATP ratios, resulting in activation of autophagy through the AMP-activated protein kinase (AMPK) pathway.^39^ The induction of autophagy can be pro-survival through the replenishment of energy supplies and degradation of the pro-apoptotic factor BNIP3.^44^ The impacts of the AMPK pathway have been confirmed with a mouse model expressing dominant-negative AMPK in cardiomyocytes, in which an attenuated autophagic response to ischemia was observed, leading to a larger AMI and worse cardiac function.^45^ However, *in vivo* glucose derived from local glycogen stores is likely to be responsible for repression of (pro-survival) autophagy. Indeed, the glycogen content in the heart is known to decrease during ischemia and be rapidly restored upon reperfusion.^10^ Furthermore, it has previously been shown in mice that cardiomyocyte death induced by IRI involves hypoxia-inducible factor (HIF)-mediated upregulation of BNIP3 and requires acidosis-induced translocation of BNIP3 to mitochondria, resulting in opening of the mitochondrial permeability transition pore (MPTP).^44,46^ We thus postulate that our *in vitro* human IRI model is in strong agreement with these previous animal studies, in which exacerbation of cardiomyocyte cell death by the provision of a low concentration of glucose at low pH during the ischemia phase is due to the suppression of ischemia-induced autophagy and BNIP3 turnover. Reduced expression of LC3II under these conditions indeed suggests that autophagy plays a protective role after only 2 h of glucose depletion during the *in vitro*-induced ischemic event. Our observation that at the physiological pH level, the addition of glucose had no significant influence on the percentage of cell death further suggests that autophagy-mediated degradation of BNIP3 is inhibited after its translocation to mitochondria by low extracellular pH, through a yet to be identified mechanism.^45,47^ This is to our knowledge the first time that such insights have been gained with a human cell-based *in vitro* model of IRI and indicate that similar mechanisms as described in mice may underlie human IRI-induced cardiomyocyte death. This important evolution of a standard IRI protocol to a more physiologically matched IRI model has thus provided new mechanistic insight and has the potential to identify new target pathways for the reduction of cardiomyocyte death post-AMI.

Our data showing that CsA effectively halts further death of human cardiomyocytes subjected to IRI are in agreement with its effectiveness as a PPC drug in animal models and human clinical trials.^8,9^ These data were recently challenged by the CIRCUS trial, which found no difference between CsA and the vehicle control group.^48^ However, the jury is clearly still undecided on CsA, as it was suggested in a subsequent publication by Heusch^49^ that this lack of difference may be related to significant differences in trial design, pretreatment of patients with P_2Y12_ antagonists (which are cardioprotective), the difference in the vehicle used [intralipid (initial trials) vs. Cremophor (CIRCUS)], and differences in the time of occlusion between these previous trials and the more recent CIRCUS trials, rather than the ineffectiveness of CsA.

Our newly established human IRI model system now allows for the rapid and cost-effective testing of some of these hypotheses and highlights its potential value. Furthermore, it can serve as an *in vitro* screening model for the discovery of novel PPC drugs and for gaining further insights into the molecular mechanisms that underlie human cardiomyocyte death/survival induced by IRI, as highlighted by our data for small molecules CHIR99021 and Gö 6976. Moreover, it may help to elucidate how the extent of cardiomyocyte death is affected by the length and level of hypoxia and reperfusion and aid in the elucidation of the exact mechanism and role of autophagy in reperfusion associated death.

Our model system is compatible with the use of iPSC-derived cardiomyocytes, permitting investigations into the role of background genotype in safety and efficacy of human PPC drugs and permitting triaging of drugs prior to costly human clinical trials. As such we anticipate that this human cell-based model system for IRI has significant potential to contribute to the discovery of new drug targets and formulations designed to increase survival and improve quality of life of humans suffering an AMI.

## METHODS

### Human pluripotent stem cell lines and culture

The use of human embryonic stem cell (HESC) H9 and human induced pluripotent stem cell (iPSC) C32 lines, along with the derivation and use thereof of reporter cells based on these lines (HESC H9-NCX1cp-GFP, iPSC C32-GCaMP6f), was under ethics approval issued by the University of Queensland Human Ethics Committee (Approval ID # 2015000667 and 2012000474). All HESC and IPSC lines were maintained in feeder-free mTESR (Stem Cell Technologies) cultures as single cell adapted cells on Matrigel™-coated plates (recommended supplier concentration). Cells were enzymatically passaged as a single cell suspension with TrypLE Express (Invitrogen). Cells were routinely karyotyped and utilized as single cells for only 0–10 passages from karyotype. For this study, H9-NCX1cp-EGFP.^26^ cells were subjected to a cardiomyocyte differentiation protocol adapted from the study by Palacek *et al.*^27^ and sorted based on GFP expression to obtain a pure cardiomyocyte population [Fig. 1(a)]. NCX1^+^-CMs formed a uniform monolayer with robust synchronous beating activity (Sup Video1).

### Generation of GCaMP6f calcium reporter cell lines

To generate a real-time intracellular Ca^2+^ lentiviral reporter, we cloned a fragment (synthesized as a GeneString by GeneArt/LifeTechnologies GmbH, Germany) containing an optimized Kozak consensus preceding an open reading frame (ORF) encoding the GCaMP6f (a fast-responding form of the GCaMP6f EGFP-based sensor^36^) using *Spe*I and *BamH*I sites into a pSIN-EF2 family lentiviral vector. The vector is optimised for constitutive transgene expression using a human EF1a promoter and contains an IRES2-puromycin resistance cassette following the transgene, allowing for an efficient selection of the transgene-expressing cells. Generation of stable clonal transgenic hESC and hiPSC lines was performed using lentiviral transduction in serum-free conditions. Briefly, the 293FT lentiviral packaging line was transfected with the pSIN-EF2-GCaMP6f-IRES-puro lentiviral vector plasmid along with pVSV-G and pCMVD8.2 plasmids, and viral particle collection was performed in a serum-free medium. Lentiviral suspensions were applied to human ES or iPS cells to generate low-density single cell-derived clones or pools by puromycin selection (2 *μ*g/ml) from post-transduction day 7. Individual clones were then assessed for pluripotency and levels of reporter expression, and robustly expressing clones or pools were used for further experimentation (directed cardiac differentiation and real-time imaging of Ca^2+^ flux in cardiomyocytes).

### Cardiomyocyte differentiation and purification protocol

Human embryonic and induced pluripotent stem cell lines were differentiated based on a protocol previously described.^26,27^ All human pluripotent stem cells were cultured in mTESR as single cells on matrigel-coated plates and seeded at 6.0 × 10^5^/cm^2^ in 6-well-plates. Daily medium exchange was performed until cells reached 100% confluence. At this point, maintenance media were removed and exchanged for cardiac induction media comprised of RPMI 1640 medium 2% B27 minus insulin (all Invitrogen) with 12 *μ*M of CHIR99021 and 300 *μ*M of Ascorbic Acid. After 24 h, media were changed to RPMI 1640 B27 without CHIR99021 and 300 *μ*M of Ascorbic Acid. After 24 h, cell media were changed into RPMI B27 minus insulin, 5 *μ*M IWP-4, and 300 *μ*M of Ascorbic Acid. At day 3, cultures were changed to basal + AA media (RPMI 2% B27 plus insulin and 300 *μ*M of Ascorbic Acid), with these media exchanged every 2 days until day 16. At day 16, cells were sorted based on NCX1cp-GFP expression. Pure cardiomyocytes were plated at a density of 5.0 × 10^5^ cells/cm^2^. Cardiomyocytes were seeded in RPMI + 2%B27 media plus insulin (all invitrogen) [Standard Media (SM)] or Dulbecco's Modified Eagle Media (DMEM), no glucose with 2% fetal calf serum (FCS), 10 mM of Galactose, 50 *μ*M of Palmitic Acid, and 100 *μ*M of Oleic acid as previously described^30^ [Fatty Acid (FA) Media]. Cells were cultured for 8 days, with media being changed every two days, before IRI modeling.

### Microgroove preparation

To drive alignment and assess mitochondria intracellular localization of cardiomyocytes matured using the FA media, aligned microgroove substrates, as reported by McDonald *et al.*,^50^ were created using standard polydimethylsiloxane (PDMS) soft lithography techniques using a SU8 on silicon master. Briefly, 100 mm silicon wafers were cleaned in an oxygen plasma and then spin coated with SU-3025 (Microchem, USA) at 500 and 3000 RPM for 10 and 30 s, respectively. The film was then processed as per the photoresist manufacturer's instructions. After spin coating, a soft bake at 95 °C was performed for 15 min. After cooling, the wafers were then exposed using an EVG-620 mask aligner (EVG, Austria) at a dose of 150 mJ/cm^2^. A post-exposure bake was performed at 65 °C for 1 min, followed by 95 °C for 5 min. These wafers were then developed in a bath of Poly(ethylene glycol) dimethacrylate (Sigma, Australia) until completion after the wafers had cooled. Groove sizes used included width to gap ratios of 75 *μ*m/25 *μ*m and 50 *μ*m/50 *μ*m, while all groves were 30 *μ*m height over several millimeters in length. Devices were then cast off the masters with Sygard 184 (Dow Corning, USA). Briefly, base and crosslinking agents were mixed at a ratio of 10:1, respectively. The PDMS mixture was then degassed to remove bubbles. Concurrently, the master was treated with Chlorotrimethylsilane (Sigma, Australia) to prevent adhesion. The PDMS mixture was then poured over the master, degassed, and baked for 20 min at 80 °C. Once cured, the PDMS pieces were cut to size, sterilized with 70% ethanol prior to use for cell culture experiments. PDMS microgrooves were coated with Matrigel (as recommended by the provider), and sorted cardiomyocytes were plated at 1.0 × 10^5^ cells/cm^2^.

### Ischemia-reperfusion *in vitro* model

NCX1^+^-CMs were plated at 1.0 × 10^5^ cells/cm^2^ in a 96 well plate format for IRI modelling. To simulate ischemia, cells were exposed for 2 h to (4-(2-hydroxyethyl)-1-piperazineethanesulfonic acid) (HEPES)-buffered medium (mM: NaCl 113; KCl 4.7, HEPES 12, MgSO_4_ 1.2, taurine 30, CaCl_2_)^43^ bubbled with nitrogen (N_2_) for 30 min, pH adjusted to 7.4 or 6.2 (hereafter Ischemia media), and incubated in a 0% oxygen incubator. For reperfusion, cell media were changed into the same HEPES buffered media, with 5.5 mM Glucose, pH adjusted to 7.4 (hereafter Reperfusion media), and incubated in a 20% oxygen incubator for 4 h. CHIR99021 and Gö 6976 were used at 1 *μ*M during reperfusion.

### Lactate dehydrogenase (LDH) release quantification

LDH release was measured as specified in the provider manual [CytoTox 96^®^ Non-Radioactive Cytotoxicity Assay (G1780)]. Briefly, after IRI, 50 *μ*l of the supernatant was carefully collected and transferred to a 96-well clear bottom plate. To each well, 50 *μ*l of substrate (CytoTox reagent) were added and plates were incubated at room temperature protected from light for 30 min. After incubation, 50 *μ*l of stop solution were added to each well and absorbance was recorded at 490 nm using a BioTek Instruments Synergy HT.

### Immunofluorescence and confocal imaging

hPSC-CM cultures were washed with phosphate-buffered saline (PBS) (Amresco) and then fixed with 4% paraformaldehyde (PFA; SIGMA; 15′, 25 °C) or kept live for mitochondrial staining. Samples were blocked/permeabilized with 10% goat serum and 0.1% Triton X (Sigma) for 5 min at room temperature. Samples were incubated with primary antibodies against cTnT 1:500 (Ab-1, Thermo Scientific) or Caspase-3 1:100 (ab32042, Abcam) for 30 min at RT. Cells were then washed with blocking buffer and incubated with secondary antibodies for 30 min (goat anti-mouse IgG1-Alexa Fluor 633 and goat anti-rabbit Alexa Fluor 647 Invitrogen). Image based analysis was used to obtain mean fluorescence intensity of Caspase-3 staining. For mitochondrial staining, Mitotracker Red FM was utilized at 100 nM for 15 min at 37 °C. For cell death quantification, cells were stained live with PI (1:1000) for 10 min followed by 10 min of fixation with 4% PFA. After fixation, cells were stained with Hoechst (1:1000) for 10 min and washed with blocking buffer.

Fluorescence was visualized using a Zeiss LSM710 laser scanning confocal microscope. Image intensities (brightness, contrast) were linearly adjusted for publication to allow clearer discernment of staining.

### Calcium flux measurement using confocal line scanning

Calcium fluorescence intensity was measured utilizing the genetically encoded calcium indicator GCaMP6f. Cardiomyocytes were incubated in Tyrode's solution (Sigma, T2397) (pH 7.4) prior to measurements and left to equilibrate for 15 min. Fluorescence measurements were performed using a Carl Zeiss LSM 710 laser scanning confocal microscope equipped with a CO_2_ and temperature control incubator. Line scanning was performed with an image size of 512 × 1 pixels, 8 bit, with a line time of 0.03 ms. All measurements were performed at 37 °C and 5% CO_2._ Calcium transients are presented as ΔF/F_0_, for background normalization, where F is the fluorescence intensity and F_0_ baseline fluorescence. A total of 20 cells per condition (N = 20) were recorded, data presented as mean ± standard error of the mean (SEM). All post-processing of the calcium imaging signals was completed in MATLAB (Mathworks, US). For each peak, the rise time (time to peak), fall time (time to baseline), rising slope, and amplitude were calculated. The rise time was determined as the time taken for the intensity to rise from 15% to local peak amplitude to the highest point of the peak. Similarly, the fall time was determined by the time it took to fall to 15% of the local peak amplitude. The rise slope was determined between 20% and 75% of the peak amplitude of each local peak through a least squares linear regression. The value of each parameter for each peak was then averaged for a given measurement.

In addition, the period and frequency were determined as a function of time. For a given signal with N peaks, N-1 values of the period and frequency were determined. The time value assigned to each of these points was the time half way between two consecutive peaks. The period was determined simply from the difference in time that each sequential peak occurred. The frequency was then determined as the inverse of the period. The period and frequency were then averaged across all points. In addition, the frequency was also confirmed representative by taking Fast Fourier Transform of the signal.

### Flow cytometry analysis

Cells were dissociated into single cells using Trypsin plus 0.25% ethylenediaminetetraacetic acid (EDTA); after incubation, each sample was split 1:1 for IgG isotype control and positive staining with anti-cTnT (Ab-1, Thermo Scientific) and anti α-actinin (A7811, Sigma-Aldrich) at RT for 30 min. Samples were next washed twice and incubated with secondary antibody (goat anti-mouse IgG-Alexa Flour 633) for 30 min at room temperature. For mitochondrial staining, Mitotracker Red FM was utilized at 100 *μ*M for 15 min at 37 °C. FACS was performed in a CFlow Accuri system, and data were analysed using CFlow Sampler Software. Multi-colour fluorescence data were acquired using well-separated acquisition channels to eliminate the need for spectral compensation. Doublets were discriminated using forward- and side-scatter pulse area, width, and height parameters. Isotype controls (mouse IgG1, Invitrogen) were used to determine non-specific staining. Cut-off levels for positive cells were set to give 1% of false positive rates based on the specific isotype control.

### Reverse transcription-quantitative polymerase chain reaction

RNA samples were collected from cultures using RNeasy Kits [including the on-column DNase treatment (Qiagen)]. RNA was quantified using a Nanodrop 1000 spectrophotometer (Thermo Scientific), and ∼350 ng of RNA was used per reaction to synthesize cDNA. cDNA synthesis was performed using a cDNA kit (Invitrogen) utilizing Superscript III for the reverse transcription. SSofast EvaGreen supermix (172-5200, Bio-Rad) was used as the master-mix for quantitative polymerase chain reactions (qPCRs). Thermocycling and analysis were performed using a CFX96 real time/C1000 thermal cycler (Bio-Rad Systems) with fast cycling parameters of 50 °C for 2 min, 95 °C for 2 min, 95 °C for 3 s, and 60 °C for 30 s for a total of 40 cycles. The results were analysed using the 2^−ΔCt^ method, calculating relative gene expression to *GAPDH*. Product efficiency was determined by melt curve analysis along with reverse transcriptase blank and water controls. Primer sequences and efficiency were validated using RNA from samples collected.

### Patch clamping

To record the spontaneous action potential of individual cells, we used a whole-cell patch-clamp configuration, as described previously.^51^ NCX1^+^-CMs+SM and FA treated cardiomyocytes were plated on Matrigel (Sigma-Aldrich)-coated glass cover slips. The patch clamp pipette solution contained (mM) 120 KCl, 1 MgCl_2_, 3 ATP, 10 HEPES, and 10 egtazic acid (EGTA) (pH 7.4). The bath recording solution contained (mM) 140 NaCl, 5.4 KCl, 1.8 CaCl_2_, 1 MgCl_2_, 10 HEPES, and 10 glucose (pH 7.4) at 36 °C (±1). Recordings of a total of 20 cells per condition were performed with whole cell capacitance compensation and changes in membrane potential being measured under the current-clamp condition (Axopatch 200A), digitized (Digidata1200 A-D converter), and recorded on a PC running pClamp8 software (Axon, Burlingame, CA, USA). Cells with ventricular-like action potential from each of the SM and FA treated populations were selected for detailed analysis.

### Statistical analysis

All data are presented as a mean ± standard error of the mean (SEM). To determine statistical differences, 2-tailed Student's *t*-tests were used with *P *<* *0.05 deemed as significant. Standard designation of confidence levels was used in figure labelling: * for p < 0.05, ** for p < 0.01, and *** for p < 0.001. For groups having the same statistical significance, the asterisk over a line covering the different conditions represents the significance between groups.

## SUPPLEMENTARY MATERIAL

See supplementary material for the Supplementary figures and videos of immature glycolytic cardiomyocytes from hiPSC that are tolerant to hypoxia (Supplementary Fig. 1), matured iPSC-derived cardiomyocytes on aligned PDMS substrates (Supplementary Fig. 2), calcium flux traces from single matured iPSC-derived GCaMP6f-cardiomyocytes (Supplementary Fig. 3), reduced expression of LC3B-II protein in matured iPSC-derived cardiomyocytes post-exposure to an ischemic event (2 h) at low pH and when glucose is present during the ischemic (hypoxia) event (Supplementary Fig. 4), iPSC-derived cardiac tissues post-induction and maturation (Supplementary Video 1), matured iPSC-derived cardiomyocytes on aligned PDMS substrates (Supplementary Video 2), and real-time calcium imaging from matured iPSC-derived GCaMP6f-cardiomyocytes (Supplementary Video 3).
